# Population Genetic Differentiation and Runs of Homozygosity Analysis of *Bursaphelenchus xylophilus* in Southwest China

**DOI:** 10.3390/genes17040443

**Published:** 2026-04-12

**Authors:** Siqi Li, Xiaoyu Li, Yuan Feng, Xiaolei Ding, Jianren Ye, Yuchen Pei

**Affiliations:** Co-Innovation Center for Sustainable Forestry in Southern China, College of Forestry, Nanjing Forestry University, Nanjing 210037, China; lisiqi@njfu.edu.cn (S.L.); lixiaoyu18@njfu.edu.cn (X.L.); yuanfeng@njfu.edu.cn (Y.F.); dxl@njfu.edu.cn (X.D.); peiyuchen@njfu.edu.cn (Y.P.)

**Keywords:** *Bursaphelenchus xylophilus*, SNP, Tibet, population genomics, whole-genome resequencing, runs of homozygosity

## Abstract

**Background**: Pine wilt disease (PWD), caused by the pine wood nematode (PWN, *Bursaphelenchus xylophilus*), is a devastating forest disease. It has been reported in five provincial-level regions in Southwest China (Chongqing, Guizhou, Sichuan, Yunnan, and Tibet), threatening local pine forest ecosystems. **Methods**: To unravel the population genetic variation and population differentiation of PWN isolates in this region, we purified eighty-one isolates for whole-genome resequencing and bioinformatics analysis, identifying candidate genes associated with runs of homozygosity (ROH). **Results**: Population structure analysis clustered the 81 isolates into three distinct genetic groups (Groups 1, 2, and 3). Notably, Group 1 exhibited fewer and shorter ROH segments compared to Groups 2 and 3, indicating higher genetic diversity and a different inbreeding history. Functional annotation of genes overlapping ROH regions revealed that Group 1 contained a subset of the genes identified in Groups 2 and 3, primarily enriched in specific molecular function categories. **Conclusions**: The PWN populations in Southwest China exhibit genetic differentiation, forming three distinct groups. Group 1 shows a reduced ROH burden and lower inbreeding levels, whereas Groups 2 and 3 display more extensive ROH patterns that may reflect historical demographic processes or potential adaptive selection. The differential distribution of ROH-associated genes across groups suggests possible variation in historical demographic processes and could suggest possible directional selection. These findings contribute to understanding the population history and genomic characteristics of PWN in Southwest China, providing insights that could support disease management strategies.

## 1. Introduction

Pine wilt disease (PWD), caused by the pine wood nematode (PWN, *Bursaphelenchus xylophilus*), is one of the most dangerous and destructive forest diseases worldwide. Native to North America [1,2], the pathogen is vectored by insects and kills pine trees rapidly by destroying vascular tissues and disrupting water transport [3]. Increasing trade and transport of timber have facilitated frequent dissemination of infected wood from endemic to non-endemic areas, accelerating the geographical expansion of the disease [4,5]. The pathogen is now established across North America [6], Europe [7], and Asia [8,9,10], with China experiencing particularly extensive infestations [11]. Data from China’s National Forestry and Grassland Administration Announcement No. 4 (2025) reveals that PWD has affected 16 provinces and municipalities, covering 620 counties and districts, thereby threatening pine forest carbon sequestration, ecosystem stability, biosecurity, and regional economies.

Southwest China, comprising Chongqing Municipality, Guizhou Province, Sichuan Province, Yunnan Province and the Tibet Autonomous Region, occupies the Yunnan-Guizhou and Qinghai–Tibet Plateaus and supports abundant pine forest resources [12]. Previous studies suggested that the region’s high elevation and lower mean annual temperatures might constrain the establishment and spread of PWN [13]. Nevertheless, research indicates that with continuous climate change, the suitable habitats for PWN in Chongqing are projected to expand [14] and field observations have demonstrated the pathogen’s presence and expansion due to anthropogenic activities [15] and unknown factors. Initial detection in Southwest China occurred in 2001 in Changshou County, Fuling District, and Wanzhou District of Chongqing. Subsequent records documented outbreaks in 2003 in Zunyi County (Zunyi City) and Xiaohe District (Guiyang City) of Guizhou Province, followed by reports in 2004 in Linshui County (Guang’an City), Sichuan Province, and Ruili City (Dehong Prefecture), Yunnan Province. Our laboratory collected infected wood from Chayu County, Nyingchi City, the Tibet Autonomous Region in 2025, marking the first detection of PWN in Tibet. This discovery extends the known distribution range of PWN to the high-altitude regions and necessitates investigation into the origin and potential adaptive mechanisms of this population. With 68 county-level administrative units currently designated as epidemic zones across the five regions, elucidating the genetic diversity, population structure and potential dispersal pathways of PWN populations in Southwest China is essential for developing targeted containment strategies.

Molecular approaches have proven valuable for investigating biological invasions, enabling analysis of pathogen genetic diversity and reconstruction of invasion routes [16,17,18]. Whole-genome resequencing facilitates comprehensive identification of genetic variants and resolution of population-level relationships [19]. Single Nucleotide Polymorphisms (SNPs) serve as high-resolution molecular markers in population genetics owing to their genomic abundance, stability, and broad distribution [20,21,22,23] compared to others [24,25]. Previous SNP-based studies of PWN have enhanced understanding of its genomic structure and evolutionary patterns [26,27,28]. Additionally, runs of homozygosity (ROH) analysis offers a quantitative framework for accessing genomic homozygosity and inbreeding. Specifically, the length and genomic distribution of ROH segments reflect demographic process and mating systems, wherein longer ROH segments indicate recent inbreeding events and diminished genetic diversity [29]. Accordingly, ROH analysis has been employed in population genetics to reconstruct demographic histories and calculate inbreeding coefficients.

Current understanding of PWN population genetics in Southwest China was not well documented in previous studies regarding other areas in China like South China and Central China [28,30]. The phylogeographic relationship between the Tibetan isolates and populations from other parts of Southwest China is crucial to understand the dispersal of pine wood nematode in this area given the high elevation and lower mean annual temperatures. Moreover, high-resolution characterization of demographic parameters, including inbreeding events, is lacking. These limitations hinder accurate reconstruction of invasion routes and impede the formulation of management protocols. To address these constraints, we performed whole-genome resequencing on 81 PWN isolates collected from Southwest China, generating SNP datasets for population analysis. This study aimed to define the population genetic structure of PWN across Southwest China and infer the demographic history of different genetic groups using ROH analysis. Furthermore, we sought to deduce the genetic provenance and probable introduction mechanism of the Tibetan population. The findings provide insights into the invasion history of PWN in this region and contribute to the development of evidence-based management strategies for PWD.

## 2. Materials and Methods

### 2.1. Isolation and Purification of Nematodes

A total of 81 isolates were collected from diseased pine wood samples from epidemic areas across five provinces in Southwest China using the Baermann funnel technique [31]. Sampling spanned the period from 2014 to 2025 and was conducted in collaboration with local forestry quarantine stations. Species identification was carried out through morphological and molecular methods following established protocols [6,32,33]. Each verified isolate was selected under a stereomicroscope and transferred onto PDA plates pre-inoculated with *Botrytis cinerea*. Cultures were incubated at 25 °C until the fungal mycelia were completely consumed (approximately 7–10 days). Subsequently, nematode suspensions were harvested and washed [28,34]. All the samples were established and maintained at the Forest Pathology Laboratory, Nanjing Forestry University. Isolates were designated using a code comprising the provincial abbreviation followed by a serial number (e.g., XZ01 for the first isolate from the Tibet Autonomous Region); detailed information is provided in Appendix A.

### 2.2. Whole-Genome Resequencing

Genomic DNA was extracted from PWNs from diverse geographical regions via cetyltrimethylammonium bromide (CTAB) method [35,36]. DNA concentration and purity were assessed with a NanoDrop 2000c spectrophotometer (Thermo Fisher Scientific, Waltham, MA, USA). All samples were quantified, normalized, and labeled with IDs corresponding to their specific nematode isolates (e.g., XZ01). High-quality DNA specimens were stored at −80 °C in the Forest Pathology Laboratory at Nanjing Forestry University before being submitted to Novogene Bioinformatics Technology Co., Ltd. (Beijing, China) for library preparation and sequencing. Paired-end libraries (150 bp) were constructed and sequenced on an Illumina HiSeq 4000 platform (San Diego, CA, USA). This process generated approximately 8 Gb of raw data per sample, achieving an average sequencing depth of >40×.

### 2.3. Identification and Filtration of Mutation Sites

Raw sequencing data quality was assessed using FastQC (v0.11.9) (http://www.bioinformatics.babraham.ac.uk/projects/fastqc/, accessed on 11 January 2026). Adapters and low-quality bases were trimmed via Cutadapt and custom PERL scripts, respectively. Cleaned reads were aligned to the reference genome [29] by utilizing the Burrows-Wheeler Aligner (v0.7.17) (BWA, http://bio-bwa.sourceforge.net, accessed on 11 January 2026). The resulting SAM files were converted to sorted BAM format, and PCR duplicates were removed by SAMtools (v1.21) (http://samtools.sourceforge.net/samtools.shtml, accessed on 11 January 2026) and Picard toolkit (v3.4.0) (http://broadinstitute.github.io/picard/, accessed on 11 January 2026). Genome-wide SNP calling was performed with FreeBayes (v1.3.10) (https://github.com/ekg/freebayes, accessed on 11 January 2026), applying a minimum coverage threshold of 10×. The resulting Variant Call Format (VCF) files were processed using VCFtools (v4.1) (http://vcftools.sourceforge.net/, accessed on 11 January 2026) and custom PERL scripts to extract key metrics, including the number of SNPs per sample, homozygous sites, private SNPs, and genotypic variations.

### 2.4. Genetic Structure Analysis

The SNPs with low allele frequency, high linkage disequilibrium and missing rate were filtered employing PLINK (v1.9) (https://www.cog-genomics.org/plink/, accessed on 11 January 2026) (--maf 0.05 --geno 0.02) and the R package SNPRelate (v3.6) (https://www.bioconductor.org/packages/release/bioc/html/SNPRelate.html, accessed on 11 January 2026) (ld.threshold = 0.3, missing.rate = 0.02, maf = 0.05). The pruned dataset was used for Principal Component Analysis (PCA). For phylogenetic analysis, SNPs were extracted via PLINK, converted to FASTA format using vcf-stats (https://pwwang.github.io/vcfstats/, accessed on 11 January 2026), and used to construct a maximum likelihood tree in MEGA (v11.0.11) (https://www.megasoftware.net/, accessed on 11 January 2026), which was further polished with ChiPlot (https://www.chiplot.online, accessed on 11 January 2026). Population structure was inferred using ADMIXTURE (v1.3) (https://dalexander.github.io/admixture/, accessed on 11 January 2026) to determine the optimal K value, with results visualized as stacked bar charts using the pophelper R package (v2.3.1) (https://www.royfrancis.com/pophelper/articles/index.html, accessed on 11 January 2026).

### 2.5. Measure of Runs of Homozygosity

Runs of homozygosity were identified for each individual in PLINK with the --homozyg command. The following criteria were applied to define ROHs: (1) a sliding window of 40 SNPs; (2) allowance for up to one heterozygous call and two missing calls per window to account for genotyping errors; (3) a minimum of 25 consecutive SNPs per ROH; (4) a minimum length of 100 kb to exclude short ROHs arising from strong linkage disequilibrium (LD); and (5) a minimum SNP density of 1 SNP per 50 kb. Default settings were used for all other parameters.

The calculated average SNP density is 4.86 SNPs/kb, representing high-density marker data. Considering that the reference genome is assembled at the contig level with the longest contig approximately 1.4 Mb, we categorize detectable ROH segments into three length classes: short (100–400 kb), medium (400–800 kb), and long (>800 kb).

### 2.6. Candidate Genes Within Runs of Homozygosity Segments

Genomic regions exhibiting reduced genetic diversity are often characterized by ROH. These highly homozygous segments may harbor targets of positive selection and reflect regions under potential selective pressure. To identify such candidates, SNP coverage within ROH regions was evaluated using R by calculating the frequency of each locus across all samples. Loci present in more than 90% of the samples were retained. The genomic coordinates of these filtered SNPs were then mapped to the reference GFF3 annotation file to determine their genomic features and extract corresponding Gene IDs. Candidate genes were subsequently annotated via the InterPro database and Gene Ontology (GO) terms, covering Biological Process, Cellular Component, and Molecular Function. Finally, the top 20 significantly enriched GO terms were identified and visualized using GraphPad Prism (v10.1) (https://www.graphpad.com/features, accessed on 12 January 2026) and Origin 2024 (https://www.originlab.com/, accessed on 12 January 2026).

## 3. Results

### 3.1. Sampling and Sequencing Quality

**Figure 1:**
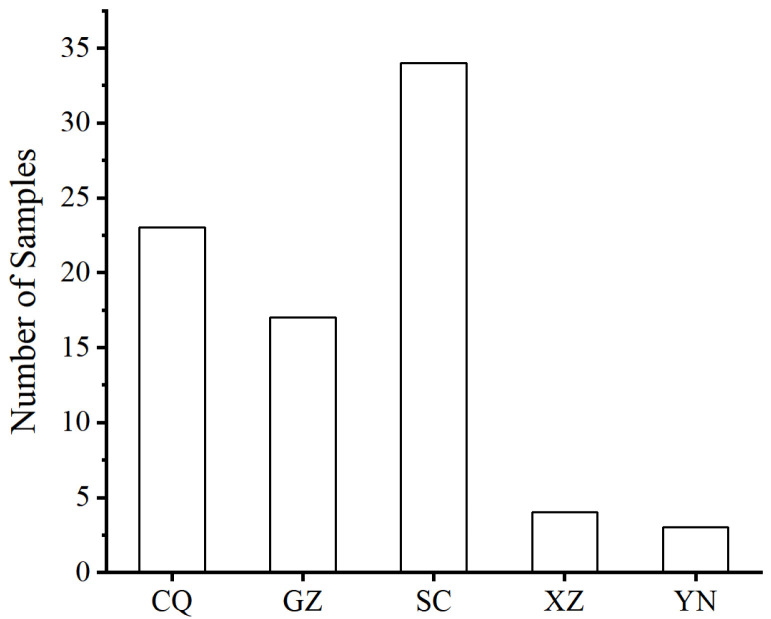
Number of samples from Chongqing (CQ), Guizhou (GZ), Sichuan (SC), Xizang (Tibet) (XZ) and Yunnan (YN).

A total of 81 PWN isolates were collected and purified across Southwest China. The isolates were collected from five regions: Chongqing Municipality (23, from 12 counties), Guizhou Province (17, from 9 counties), Sichuan Province (34, from 20 counties), Yunnan Province (3, from 2 counties) and the Tibet Autonomous Region (4, from 1 county), (Figure 1). Detailed information is provided in Appendix A.

To comprehensively assess the genetic diversity of PWNs in Southwest China, we performed deep whole-genome resequencing on the 81 isolates, generating approximately 966.7 GB of data. The sequencing quality was high, with an average sequencing depth of 111X, a mean mapping rate of 93.40%, an error rate below 0.04%, and Q30 values exceeding 82.70%, all within normal ranges, thus permitting downstream analyses. Following data filtering, a total of 9,858,218 SNP loci were identified.

### 3.2. SNP Variation

**Figure 2:**
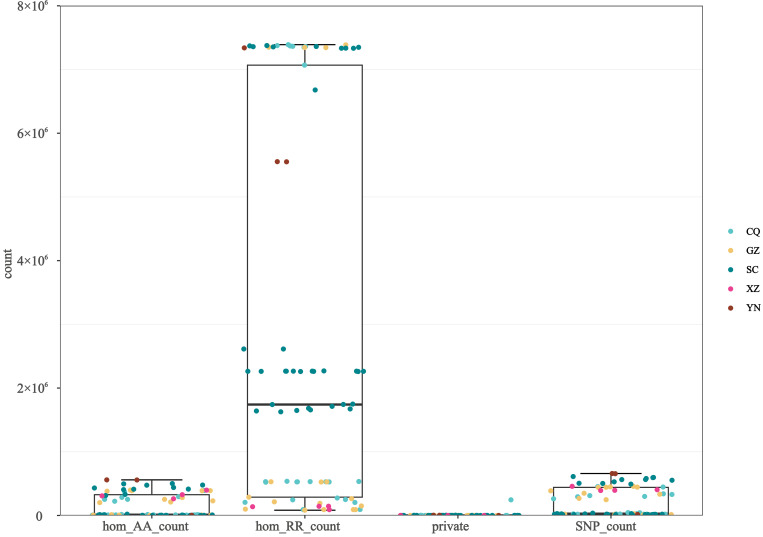
Counts of homozygous alternative alleles, homozygous reference alleles, private SNPs, and total SNPs across the 81 isolates.

Among the regions sampled, Chongqing exhibited the lowest average number of SNPs (134,720) and the lowest count of homozygous alternative alleles (96,516); however, it possessed the highest number of private SNPs (11,328). In contrast, Yunnan displayed the highest average counts for homozygous reference alleles (6,148,269), homozygous alternative alleles (373,237), and SNP count (443,772), along with an exceptionally high number of private SNPs (2494). Isolates from the Tibet Autonomous Region possessed very few private SNPs (303). Notably, the Tibetan isolates exhibited a high number of SNPs overall (412,182), yet the count of homozygous reference alleles (129,349) was lower than that of homozygous alternative alleles (323,338) (see Table 1 and Figure 2).

**Figure 3:**
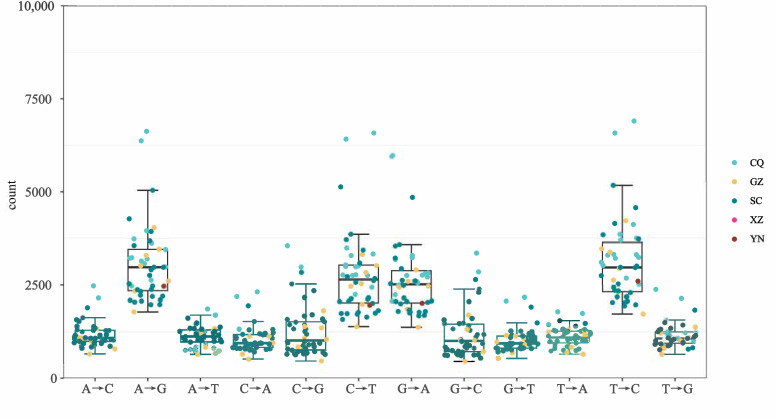
Statistical distribution of the 12 SNP genotypes across the 81 isolates.

A total of 12 SNP genotypic classes were identified across all samples (Figure 3): A→C, A→G, A→T, C→A, C→G, C→T, G→A, G→C, G→T, T→A, T→C, and T→G. The distribution of these mutation types was significantly skewed, with transitions (A→G, C→T, G→A, T→C) occurring at a markedly higher frequency than transversions. Isolates from Yunnan and Tibet exhibited significantly higher numbers of SNP genotypes compared to those from other provinces, whereas isolates from Chongqing displayed the lowest genotypic diversity.

### 3.3. Analysis of Population Genetic Structure

**Figure 4:**
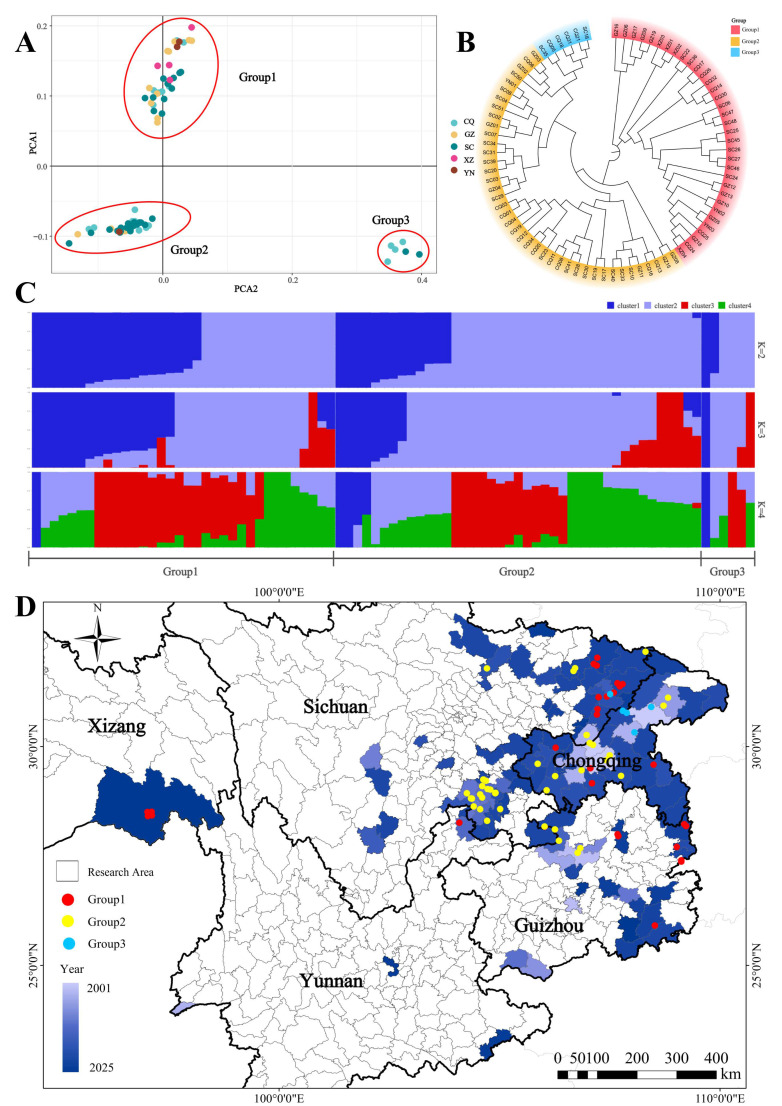
Population genetic analysis results of 81 isolates. (**A**) Principal Component Analysis (PCA). (**B**) Phylogenetic tree. (**C**) ADMIXTURE plot illustrating genetic admixture proportions with the best-fit model at K = 3. (**D**) Geographical distribution of different genetic groups.

After filtering high-quality SNP loci, we performed population structure analysis using these genetic markers. Principal Component Analysis (PCA), phylogenetic tree construction, and ADMIXTURE clustering consistently revealed that the 81 isolates could be differentiated into three distinct genetic Groups (Group 1, Group 2, and Group 3) (Figure 4).

Group 1 comprised 34 isolates collected from all five surveyed provinces: Chongqing (7), Guizhou (10), Sichuan (11), Yunnan (2), and Tibet (4). All four isolates from the newly affected area of Tibet belonged exclusively to Group 1. Group 2 was the largest Group, containing 41 isolates from four provinces: Chongqing (12), Guizhou (7), Sichuan (21), and Yunnan (1). Group 3 was the smallest, consisting of only six isolates restricted to Chongqing (4) and Sichuan (2).

A different geographical pattern emerged regarding the timing of disease outbreaks. Isolates in Group 1 (including all the Tibetan samples) were predominantly found in regions with more recent epidemic outbreaks, whereas isolates from long-established epidemic areas, such as Wanzhou District (Chongqing) and Zunyi City (Guizhou), were primarily assigned to Groups 2 and 3.

Group 1 exhibited exceptionally high genetic variation, with a total SNP count of 450,029 and 361,868 homozygous alternative alleles (Table 2). Group 2 and Group 3 demonstrated a reduction in genetic diversity, characterized by a high proportion of homozygous reference alleles (4,253,932 and 3,090,603, respectively) and significantly fewer SNPs (27,920 and 19,164). Furthermore, Group 2 possessed a large number of unique variants (6654 private SNPs).

### 3.4. Genomic Distribution of Runs of Homozygosity

To investigate the inbreeding history and genetic background of PWN populations in Southwest China, we performed a genome-wide Runs of Homozygosity analysis on the 81 isolates. A total of 6712 ROH segments were identified across all samples, with a mean total length of approximately 23 Mb per individual.

**Figure 5:**
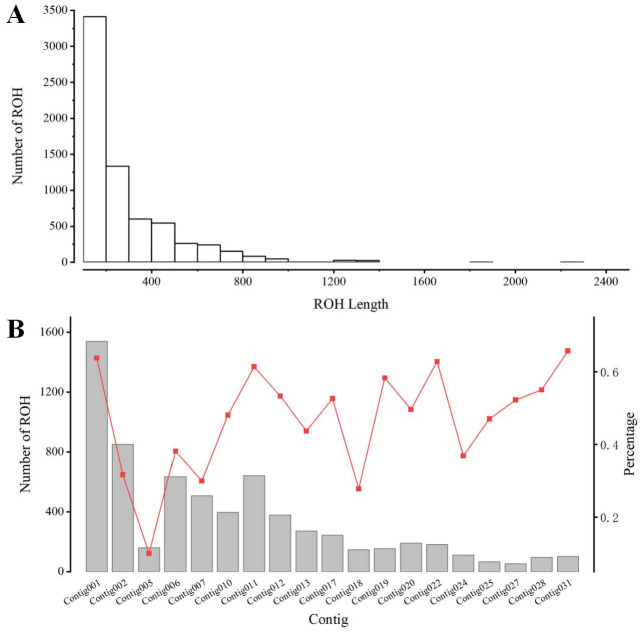
Distribution of ROH segments. (**A**) Distribution of ROH in different lengths (kb). (**B**) Number of ROH longer than 100 kb per contig (bars) and average percentage of each contig covered by ROH (red line).

The distribution of ROHs categorized by length is illustrated in Figure 5A. The ROH landscape was predominantly characterized by an abundance of short fragments, which accounted for approximately 80% of the total number of detected ROHs and contributed about 60% of the cumulative ROH length. In contrast, long ROH segments, although representing only about 3% of the total count, covered approximately 10% of the cumulative ROH length.

At the contig level, the number of ROHs and the percentage of genomic coverage varied significantly among contigs (Figure 5B). The highest number of ROHs was observed on contig001 (1538 segments), whereas the lowest was found on contig027 (53 segments). On average, 29.6% of the genome was covered by ROHs. Coverage rates ranged from a maximum of 66% on contig031 to a minimum of 10% on contig003.

We calculated the proportion of the genome covered by ROHs (F_ROH_) for each group. Group 1 exhibited the lowest F_ROH_, averaging approximately 7%. Groups 2 and 3 showed higher inbreeding levels, with an average F_ROH_ of approximately 46%. Within Groups 2 and 3, short, medium, and long ROH segments contributed roughly 23%, 18%, and 5% to the total genome coverage, respectively. Statistical analysis revealed that the F_ROH_ of Group 1 was significantly lower than that of Groups 2 and 3 (*p* < 0.05), while no significant difference was observed between Groups 2 and 3.

**Figure 6:**
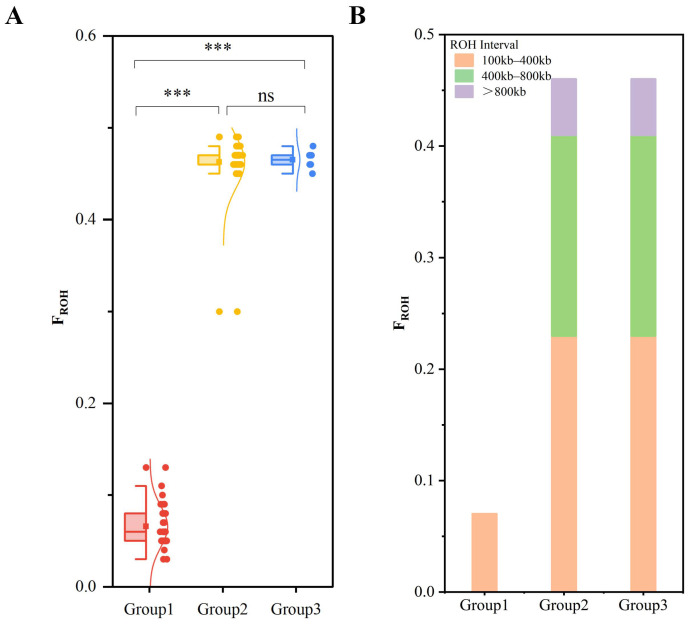
ROH analysis across different groups. (**A**) Significance test of differences in F_ROH_; (**B**) Statistical composition of ROH segment types (*** *p* < 0.001, ns = not significant, *p* > 0.05).

Further analysis of the ROH composition within each group indicated different patterns. Isolates in Group 1 possessed the fewest total ROH segments (1178) and were characterized by short fragments. Conversely, isolates in Groups 2 and 3 displayed a diverse composition containing all three length categories. Specifically, these groups contained 1037 and 153 medium segments and 160 and 21 long segments, respectively (Figure 6; detailed data in Table 3).

### 3.5. Candidate Genes Within Runs of Homozygosity

We extracted SNPs located within the 6712 identified ROH segments, yielding a total of 15,960,387 raw SNPs. After filtering for a genotype call rate of >90% within each group, we retained 4984, 330,522, and 329,944 high-quality SNPs for Group 1, Group 2, and Group 3, respectively. Annotation of these SNPs demonstrated variation in the number of associated genes: Group 1 contained only 122 genes, Groups 2 and 3 harbored 2193 and 2176 genes, respectively.

**Figure 7:**
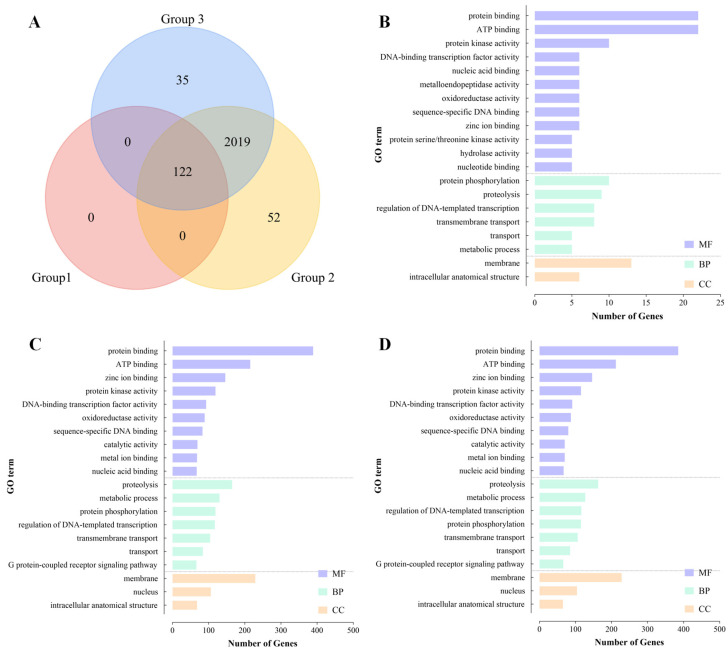
Number of candidate genes across different genetic groups (**A**) and Gene Ontology (GO) annotation results: (**B**) Group 1, (**C**) Group 2, and (**D**) Group 3.

Venn diagram (Figure 7A) showed that all 122 genes in Group 1 were shared among all three Groups. Groups 2 and 3 shared a large core set of 2019 genes, while possessing 52 and 35 Group-specific unique genes, respectively.

Functional enrichment analysis was performed by mapping candidate gene IDs to the InterPro database and associated Gene Ontology (GO) terms. The top 20 most frequent GO terms for each group are illustrated in Figure 7B–D. Across all groups, the mostly enriched terms were concentrated in the Molecular Function category, notably ‘protein binding’, ‘ATP binding’, and ‘zinc ion binding’. Additionally, ‘protein kinase activity’ and ‘DNA-binding transcription factor activity’ were consistently enriched in all groups.

Both Group 2 and Group 3 showed enrichment in the ‘G protein-coupled receptor (GPCR) signaling pathway’. As a core mechanism for cellular environmental sensing and response via second messenger systems, this pathway enables precise regulation of physiological activities. The presence of extensive ROHs containing these specific genes in Groups 2 and 3 suggests that these populations may have undergone potential directional selection, leading to the fixation of alleles beneficial for adapting to their current local environments. The scarcity of ROH-associated genes in Group 1 indicates that isolates in this group probably have retained higher genetic diversity.

## 4. Discussion

This study provides the first comprehensive genomic characterization of PWN populations in Southwest China. Serving as the core foundation for species evolution, high SNP abundance indicates a great variety of genetic variations [37,38]. While the founder effect is typically associated with a loss of genetic diversity, multiple introduction events can restore or even enhance it [39]. We identified 9,858,218 SNP loci across 81 samples, which exceeds that reported in related studies [28,30,40], indicating that PWNs in Southwest China possess richer genetic diversity.

We identified three distinct genetic groups based on principal component analysis, phylogenetic tree and ADMIXTURE analyses. Group 1 exhibited higher genetic diversity and lower inbreeding levels, as indicated by a high number of SNPs as well as fewer and shorter ROH segments. This pattern suggests that isolates in Group 1 may has not experienced intense directional selection yet or possess a more complex and diverse genetic background, showing low inbreeding and high diversity which may confer broader genetic plasticity to Group 1 and endow it with enhanced evolutionary potential to cope with unpredictable environments [41].

The newly discovered four Tibetan isolates collected in 2025 were exclusively assigned to Group 1 and displayed a different genotypic pattern characterized by a higher proportion of homozygous alternative alleles compared to reference alleles. This pattern suggests that the Tibetan population might originate from a genetically diverse source different from the reference strain (derived from Jiangsu-Anhui region). The high genetic variation and absence of severe bottlenecks in the Tibetan population contrast with typical invasion patterns and may support the hypothesis of multiple independent introduction events rather than a single recent invasion [37,38,39]. While Tibet’s high altitude and low temperatures generally act as natural barriers against nematode invasion [42,43], low-altitude, warm, and humid regions in the southeast, such as Nyingchi and Gyirong County, remain at risk. These areas feature annual mean temperatures of 8–13 °C, abundant precipitation, extensive pine forests [44], and the presence of the vector beetle *Monochamus alternatus* [45]. Moreover, given PWN’s genetic basis for cold adaptation [40] and rapid evolutionary potential, human-mediated transport via timber [46] could enable the nematode to adapt to local conditions and spread. Coincidentally, the samples obtained in this study originated from Nyingchi, Tibet. This suggests that without strict control measures, the PWD epidemic in southeastern Tibet could worsen significantly.

Groups 2 and 3 showed increased inbreeding, suggesting different demographic histories. The consistency of our clustering of geographically distant isolates with previous studies [47] suggests active gene flow facilitated by human-mediated transport of infested wood, highlighting the role of anthropogenic factors in pathogen dispersal. The source locations of isolates in Groups 2 and 3 encompass some of the earliest recorded epidemic sites in Southwest China, specifically Zunyi County, Guizhou (Group 2, isolate GZ01) and Wanzhou District, Chongqing (Group 3, isolate CQ05). This spatial distribution suggests that these two groups probably represent the earliest established and subsequently disseminated lineages in the region. Group 3 comprises only four isolates from Chongqing and two from Sichuan. Specifically, the Yunnan isolates in our study showed unusually high genetic richness and were distributed across Groups 1 and 2, suggesting that Yunnan populations may have different origins and some may originate from Sichuan, consistent with the conclusions of Ding et al. [47], which clustered one Yunnan isolate together with Sichuan isolates.

In addition, Group 1 represents a genetically diverse population with a high number of alternative alleles, resulting in a large total SNP count. Groups 2 and 3 are characterized by extensive genomic homozygosity, where the vast majority of loci match the reference genome. This indicates a loss of genetic variation in these groups, leading to a very low number of detectable SNPs. This pattern is consistent with the ROH analysis.

ROH analysis revealed different inbreeding histories among the three genetic groups. Groups 2 and 3 showed enrichment of genes involved in GPCR signaling pathways, ATP binding, and protein kinase activity within their ROH regions. These functional categories are critical for environmental sensing, energy metabolism, and stress response [48,49]. In opposition, Group 1 exhibited fewer and shorter ROH segments with minimal functional enrichment, indicating either a more complex genetic background or the absence of intense recent selection. This “low inbreeding and high diversity” state may confer greater evolutionary potential to Group 1, enabling more potential flexible responses to future environmental challenges.

The genetic heterogeneity observed across Southwest China has important implications for pine wilt disease management. The distinct genetic composition of the Tibetan population suggests that this region may require specific quarantine considerations. Management strategies should account for regional genetic diversity and the complex introduction history revealed by our analyses. The absence of historical samples and environmental data integration also restricts temporal and ecological interpretations. Nevertheless, our results offer genomic insights for pine wilt disease management.

## 5. Conclusions

This study provides the first comprehensive genomic characterization of PWN populations in Southwest China, identifying three genetic groups across 81 samples, among which homozygosity levels varied largely. The newly discovered Tibetan isolates belong to Group 1 exclusively and display a distinct genotypic pattern dominated by homozygous alternative alleles, indicating a genetically diverse source distinct from the reference strain and likely resulting from complex, multiple introduction pathways. Several genes within ROH segments enriched in GPCR signaling pathways in Groups 2 and 3, which may reflect that historical demographic processes should be the subject of further investigation for potential adaptive significance. These findings may support the implementation of quarantine measures in Tibet and suggest that management strategies must account for the region’s high genetic heterogeneity and diverse invasion history.

## Figures and Tables

**Table 1:** Genetic diversity statistics of PWN isolates by province.

Province	Hom. Alt. Alleles	Hom. Ref. Alleles	Private SNPs	SNP Count
CQ	96,516	2,496,080	11,328	134,720
GZ	186,506	1,916,066	511	232,500
SC	141,282	3,591,603	714	192,046
XZ	323,338	129,349	303	412,182
YN	373,237	6,148,269	2494	443,772

**Table 2:** Genomic statistics for each group.

Group	Hom. Alt. Alleles	Hom. Ref. Alleles	Private SNPs	SNP Count
Group 1	361,868	1,120,730	804	450,029
Group 2	6504	4,253,932	6654	27,920
Group 3	6159	3,090,603	345	19,164

**Table 3:** Summary of ROH statistics for different groups.

	Number	Length (Mb)	Mean Length (Mb)	Category	Number	Length (Mb)	Mean Length
Group 1	1178	172.39	0.15	Short	1178	172.39	0.15 ± 6.24
				Medium	0	0	0
				Long	0	0	0
Group 2	4828	1469.1	0.30	Short	3631	742.32	0.20 ± 5.25
				Medium	1037	566.64	0.55 ± 11.82
				Long	160	160.14	1.00 ± 60.57
Group 3	706	215.27	0.30	Short	532	107.81	0.20 ± 4.60
				Medium	153	84.35	0.55 ± 10.89
				Long	21	23.11	1.10 ± 132.98

## Data Availability

The original contributions presented in this study are included in this article; further inquiries can be directed to the corresponding author.

## Appendix A

**Table A1:** Geographic origins of 81 PWN isolates.

ID	Host	Geographic Origin	Sampling Date	Year of First Report
CQ01	Unknown	Fuling District, Chongqing	Nov. 2014	2001
CQ03	*Pinus massoniana*	Banan District, Chongqing	Jan. 2015	2004
CQ04	*P. massoniana*	Yunyang County, Chongqing	Jul. 2015	2006
CQ05	*P. massoniana*	Wanzhou District, Chongqing	Jul. 2015	2001
CQ06	*P. massoniana*	Wanzhou District, Chongqing	Jul. 2015	2001
CQ08	*P. massoniana*	Fuling District, Chongqing	Sep. 2016	2001
CQ11	*P. massoniana*	Yunyang County, Chongqing	Sep. 2016	2006
CQ12	*P. massoniana*	Changshou District, Chongqing	Oct. 2024	2001
CQ13	*P. massoniana*	Jiangjin District, Chongqing	Oct. 2024	2017
CQ14	*P. massoniana*	Fuling District, Chongqing	Oct. 2024	2001
CQ16	*P. massoniana*	Jiangjin District, Chongqing	Oct. 2024	2017
CQ17	*P. massoniana*	Wulong District, Chongqing	Oct. 2024	2018
CQ18	*P. massoniana*	Liangping District, Chongqing	Oct. 2024	2018
CQ19	*P. massoniana*	Changshou District, Chongqing	Oct. 2024	2001
CQ20	*P. massoniana*	Wulong District, Chongqing	Oct. 2024	2018
CQ21	*P. massoniana*	Zhongxian County, Chongqing	Oct. 2024	2004
CQ24	*P. massoniana*	Hechuan District, Chongqing	Oct. 2024	2019
CQ25	*P. massoniana*	Hechuan District, Chongqing	Oct. 2024	2019
CQ26	*P. massoniana*	Pengshui Miao and Tujia Autonomous County, Chongqing	Oct. 2024	2019
CQ30	*P. massoniana*	Nanchuan District, Chongqing	Oct. 2024	2018
CQ31	*P. massoniana*	Zhongxian County, Chongqing	Oct. 2024	2004
CQ32	*P. massoniana*	Nanchuan District, Chongqing	Oct. 2024	2018
CQ34	*P. massoniana*	Wulong District, Chongqing	Oct. 2024	2018
GZ01	Unknown	Bozhou District, Zunyi City, Guizhou Province	Dec. 2014	2017
GZ02	Unknown	Bozhou District, Zunyi City, Guizhou Province	Dec. 2014	2017
GZ03	*P. massoniana*	Renhuai City, Zunyi City, Guizhou Province	Dec. 2014	2014
GZ04	*P. massoniana*	Renhuai City, Zunyi City, Guizhou Province	Jul. 2015	2014
GZ05	Unknown	Songtao Miao Autonomous County, Tongren City, Guizhou Province	Oct. 2024	2020
GZ06	*P. massoniana*	Wanshan District, Tongren City, Guizhou Province	Oct. 2024	2019
GZ08	*P. massoniana*	Xishui County, Zunyi City, Guizhou Province	Oct. 2024	2019
GZ09	*P. massoniana*	Xishui County, Zunyi City, Guizhou Province	Oct. 2024	2019
GZ10	*P. massoniana*	Rongjiang County, Qiandongnan Miao and Dong Autonomous Prefecture, Guizhou Province	Oct. 2024	2020
GZ11	Unknown	Renhuai City, Zunyi City, Guizhou Province	Oct. 2024	2014
GZ12	*P. massoniana*	Fenggang County, Zunyi City, Guizhou Province	Oct. 2024	2021
GZ13	*P. massoniana*	Fenggang County, Zunyi City, Guizhou Province	Oct. 2024	2021
GZ15	*P. massoniana*	Xishui County, Zunyi City, Guizhou Province	Oct. 2024	2019
GZ16	*P. massoniana*	Bijiang District, Tongren City, Guizhou Province	Oct. 2024	2018
GZ17	*P. massoniana*	Bijiang District, Tongren City, Guizhou Province	Oct. 2024	2018
GZ18	*P. massoniana*	Songtao County, Tongren City, Guizhou Province	Oct. 2024	2020
GZ19	Unknown	Wanshan District, Tongren City, Guizhou Province	Oct. 2024	2019
SC02	*P. massoniana*	Gaoxian County, Yibin City, Sichuan Province	Mar. 2015	2013
SC03	*P. massoniana*	Fushun County, Zigong City, Sichuan Province	Mar. 2015	2013
SC04	*P. massoniana*	Cuiping District, Yibin City, Sichuan Province	Mar. 2015	2012
SC06	*P. massoniana*	Tongchuan District, Dazhou City, Sichuan Province	Mar. 2015	2015
SC07	*P. massoniana*	Yibin County, Yibin City, Sichuan Province	Mar. 2015	2011
SC08	*P. massoniana*	Pingshan County, Yibin City, Sichuan Province	Nov. 2015	2016
SC10	*P. massoniana*	Pingshan County, Yibin City, Sichuan Province	Nov. 2015	2016
SC17	*P. massoniana*	Jiangyou City, Mianyang City, Sichuan Province	Aug. 2017	2017
SC18	*P. massoniana*	Dachuan District, Dazhou City, Sichuan Province	Mar. 2019	2017
SC19	*P. massoniana*	Bazhou District, Bazhong City, Sichuan Province	Mar. 2019	2019
SC20	*P. massoniana*	Ziliujing District, Zigong City, Sichuan Province	Aug. 2022	2018
SC22	*P. massoniana*	Tongjiang County, Bazhong City, Sichuan Province	Sep. 2022	2020
SC23	*P. massoniana*	Linshui County, Guang’an City, Sichuan Province	Sep. 2022	2004
SC24	*P. massoniana*	Tongjiang County, Bazhong City, Sichuan Province	Sep. 2022	2020
SC25	*P. massoniana*	Dazhu County, Dazhou City, Sichuan Province	Sep. 2022	2018
SC26	*P. massoniana*	Xuanhan County, Dazhou City, Sichuan Province	Sep. 2022	2019
SC27	*P. massoniana*	Xuanhan County, Dazhou City, Sichuan Province	Sep. 2022	2019
SC28	Unknown	Enyang District, Bazhong City, Sichuan Province	Sep. 2022	2019
SC29	*P. massoniana*	Ziliujing District, Zigong City, Sichuan Province	Aug. 2022	2018
SC30	*P. massoniana*	Linshui County, Guang’an City, Sichuan Province	Sep. 2022	2004
SC31	*P. massoniana*	Nanxi District, Yibin City, Sichuan Province	Sep. 2022	2018
SC33	*P. massoniana*	Gong County, Yibin City, Sichuan Province	Sep. 2022	2019
SC34	*P. massoniana*	Nanxi District, Yibin City, Sichuan Province	Sep. 2022	2018
SC35	*P. massoniana*	Kaijiang County, Dazhou City, Sichuan Province	Sep. 2022	2021
SC36	*P. massoniana*	Tongjiang County, Bazhong City, Sichuan Province	Sep. 2022	2020
SC39	*P. massoniana*	Cuiping District, Yibin City, Sichuan Province	Sep. 2022	2012
SC40	*P. massoniana*	Changning County, Yibin City, Sichuan Province	Sep. 2022	2019
SC41	*P. massoniana*	Gaoxian County, Yibin City, Sichuan Province	Sep. 2022	2013
SC45	Unknown	Dachuan District, Dazhou City, Sichuan Province	Sep. 2022	2017
SC46	Unknown	Dachuan District, Dazhou City, Sichuan Province	Sep. 2022	2017
SC47	*P. massoniana*	Dazhu County, Dazhou City, Sichuan Province	Sep. 2022	2018
SC48	Unknown	Tongchuan District, Dazhou City, Sichuan Province	Oct. 2022	2015
SC50	*P. massoniana*	Wanyuan City, Dazhou City, Sichuan Province	Sep. 2022	2019
SC51	*P. massoniana*	Fushun County, Zigong City, Sichuan Province	Sep. 2022	2013
XZ01	*Pinus yunnanensis*	Zayu County, Nyingchi City, Xizang Autonomous Region	May 2025	2025
XZ02	*P. yunnanensis*	Zayu County, Nyingchi City, Xizang Autonomous Region	May 2025	2025
XZ03	*P. yunnanensis*	Zayu County, Nyingchi City, Xizang Autonomous Region	May 2025	2025
XZ04	*P. yunnanensis*	Zayu County, Nyingchi City, Xizang Autonomous Region	May 2025	2025
YN01	*P. massoniana*	Shuifu County, Zhaotong City, Yunnan Province	Jan. 2015	2019
YN02	*P. yunnanensis*	Zhaotong City, Yunnan Province	Oct. 2021	2013
YN03	*P. yunnanensis*	Zhaotong City, Yunnan Province	Oct. 2021	2013
